# The smartphone as a “significant other”: interpersonal dependency and attachment in maladaptive smartphone and social networks use

**DOI:** 10.1186/s40359-023-01339-4

**Published:** 2023-09-28

**Authors:** Emanuela S. Gritti, Robert F. Bornstein, Baptiste Barbot

**Affiliations:** 1 Department of Psychology, Milano-Bicocca University, Piazza dell’Ateneo Nuovo 1, Milan, 20126 Italy; 2 Derner Institute of Advanced Psychological Studies, Adelphi University, Garden City, USA; 3 UCLouvain, Psychological Sciences Research Institute, Louvain, Belgium

**Keywords:** Smartphone use, Social networking, Attachment style, Interpersonal dependency, Human-computer interaction

## Abstract

Smartphones are increasingly widespread throughout the world and, although smartphones provide various benefits, excessive and maladaptive use is often reported. Given the penetration of smartphones in the individual’s daily life, it is relevant to identify the mechanisms sustaining their use, including the affective bond that the owner may develop with the device. The aim of the current study is to test a novel model to explain smartphone and Social Network Sites (SNS) use from an interpersonal perspective. We hypothesized that adult attachment style and interpersonal patterns (i.e., features of interpersonal dependency) generalize to the emotional bond with the mobile device, interacting with psychological correlates and background factors to predict smartphone and SNS consumption. 341 nonclinical adults (57.2% females; age M = 35.5, SD = 14.6) completed a battery consisting of the Attachment Style Questionnaire, the Relationship Profile Test, the Rosenberg Self-Esteem Scale, the Toronto Alexithymia Scale, the Young Adult Attachment to Phone Scale, and the Social Network Intensity and Social Network Access via Mobile phone Applications. A multi-mediation model supported the hypothesis regarding the influence of interpersonal style in the relationship of the individual with their smartphone and use of SNS. A parallel between attachment style and the emotional bond with the smartphone emerged, with anxious attachment style and destructive overdependence being potential risk factors for maladaptive smartphone use especially in individuals involved in a romantic relationship. Findings are discussed in terms of theoretical implications and intervention strategies towards smartphone dependency.

**Supplementary Information:**

The online version contains supplementary material available at 10.1186/s40359-023-01339-4.

## Introduction

Smartphones are ubiquitous in everyday life, especially in technologically more advanced societies, with a median reported ownership rate of 76%, spanning from 95% in South Korea to 59% in Greece [1). Mobile phones have the potential for several psychosocial benefits [2, 3], but also pose risks for mental health such as the incapacity to regulate the use of the device in a flexible and adaptive way, also labeled “Problematic Mobile Phone Use” (PMPU; see [4, 5]). Psychological factors contributing to maladaptive smartphone use have been investigated, although so far, the topic has been mostly approached using an addiction model that extends to maladaptive use of technology. Several authors outlined the limits of such an approach, which oversimplifies the complex and multifaceted smartphone use behavior, ignoring key psychological processes involved in both its adaptive and maladaptive aspects [5, 6].

In particular, a novel line of research exploring the associations between adult attachment styles and relationship with one’s smartphone is emerging [7–9]. These studies focused mainly on the prediction of problematic smartphone use based on the individual’s attachment style, highlighting anxious attachment as a relevant factor in sustaining smartphone addiction [10]. Although these contributions are important, they have not examined whether adult attachment styles actually transfer to the relationship with one’s smartphone and if there are specific psychological functions fulfilled by the device for the individual. In particular, little is known about the potential self-regulatory function that the smartphone (and its use) might have for insecurely attached individuals, similar to the role played by significant others. Further, only limited attention has been given so far to the putative mediators (e.g., low self-esteem and emotional dysregulation including alexithymia) through which adult attachment could affect smartphone use [11, 12].

The theoretical framework of smartphone use (and misuse) can be further enriched by expanding the analysis of individual interpersonal characteristics to another relevant dimension: interpersonal dependency [13]. The latter can be defined as the capacity to form relationships with others characterized by mutual support and adequate boundaries (i.e., healthy dependency), versus excessively relying on others for guidance and nurturance (i.e., destructive overdependence) or failing to connect with others, remaining socially disconnected and aloof (i.e., detachment). Interpersonal dependency appears promising in helping understanding the emotional bond that users develop with their smartphone also given the associations that maladaptive interpersonal dependency has with other forms of addiction [14], as well as with risk factors for digital addiction itself such as depression, low self-esteem, and alexithymia [15–17].

By facilitating access to Social Networking Sites (SNS) and messaging apps, smartphones allow people to *communicate* and *connect*, helping to satisfy crucial psychological needs. Increased interpersonal communication through smartphones can reinforce social bonds across the various domains of interpersonal relationships [3, 18]. However, this “interactive” feature of smartphones also constitutes a documented risk factor for smartphone addiction. Studies conducted across different cultures consistently show that identifying social networking as the most personally relevant function of the smartphone, and reporting high SNS intensity of use, are significant predictors of smartphone addiction [19, 20]. As such, the present study extends the focus beyond the individual relationship with the smartphone (i.e., smartphone attachment) to include the intensity of SNS use.

### Conceptualizing the bond with one’s smartphone in an interpersonal perspective: attachment styles and interpersonal dependency

Based on concepts and findings from object relations theory [21] and attachment theory [22] we propose that a critical, yet neglected, motive for smartphone use involves the interpersonal and attachment patterns of the individual, especially one’s propensity to connect to, rely, and ultimately “depend” on others [13, 23]. Notably, a contemporary line of research has shown that individual differences pertaining to attachment styles, in particular anxious attachment, can represent a risk factor for problematic use of social networks [24]. This can happen because users using SNS primarily for maintaining and developing social relationships might differ in the way they use these sites based on their attachment style. In addition, it’s possible that individuals with insecure attachment, who often developed less adequate emotion regulations capacities [25, 26], more frequently revert to a compulsive use of SNS to cope with unpleasant emotional experiences. A link between adult insecure attachment style and the tendency to recur to external forms of emotional regulation have been documented for example in the case of both substance [26] and digital addiction [24]. Along these lines, dependent personality functioning (i.e., the presence of a Dependent Personality Disorder diagnosis) has been identified as a worsening factor for patients with alcohol and substance abuse patients [27]. However, no study has investigated the role of interpersonal dependency, conceived in its adaptive (i.e., healthy dependency) and maladaptive variants (i.e., overdependence and detachment) in the context of maladaptive smartphone and SNS use.

Examining both the attachment-based and interpersonal (i.e., dependency-related) dynamics of smartphone use is particularly important in this context because prior research has shown that attachment-related behaviors tend to be exhibited selectively and directed toward certain individuals and not others, whereas dependency-related behaviors are exhibited less selectively, and in a broader range of contexts and situations [28, 29]. In addition, studies have shown that in many interpersonal contexts information regarding a person’s underlying and expressed dependency needs adds incremental validity—unique predictive value—in behavioral prediction, relative to investigations that assess only attachment styles [30].

### The excessive reassurance pathway to smartphone use: smartphones as transitional objects

As posited by the “excessive reassurance pathway” to smartphone use [5], it is likely that the use of the device, especially when particularly intense, is predominantly driven by the necessity to maintain relationships and gain reassurance from others, and the behavior is reinforced by individual factors such as insecure attachment, emotional instability, and low self esteem. However, recent evidence indicates that people can emotionally attach to their phone regardless of the opportunity it offers to reach out to significant others: smartphones can also be used to compensate the perceived unavailability of attachment figures notwithstanding their ability to facilitate communication with them [31]. This is in line with the idea rooted on object relations theory that inanimate objects can perform a transitional function in adult age, beyond childhood, providing relief and comfort especially in moments of distress [31–34] or for people with heightened psychological vulnerability (e.g., pathological hoarders; [35]).

Several unique features of the smartphone make it particularly suitable as an attachment target. It is highly controllable and constantly available, customizable, and able to provide a “tactile” experience [7]. Moreover, as portable devices, smartphones are small or lightweight enough to be carried around for use across different contexts and settings [36]. In addition, because smartphones are typically personalized by the owner to provide a relatively effortless way to interact with valued others, they represent a “direct” connection to absent objects. To the extent that a device (like a smartphone) is strongly linked with features of the absent individual in the mind of the person using that device, it has the potential to evoke emotional responses that are particularly powerful and compelling [37, 38].

### Contrasting patterns of smartphone use

Although the “transitional” function that inanimate objects can serve beyond infancy has long been documented [39–41], conceptualizing the relationship with smartphones as an attachment relationship has only been suggested more recently. Investigations that have gone beyond the construct of attachment styles as predictors of maladaptive smartphone use have highlighted the existence of two different antithetic, and emotionally loaded relationship patterns that users may form with their smartphone [42, 43]. A large-scale study on U.S. adults documented two polarized views of the device that users may have [44]. When forced to choose an option among two contrasting alternatives, 54% of participants identified the device as something that they “couldn’t live without”, with the remaining stating the opposite. Along the same lines, 70% of the respondents described the phone as a source of “freedom” whereas the remaining 30% identified it as “a leash”. Building upon this literature, in a large (N = 955) community sample of individuals from the U.S. aged 18–29 years, Trub and Barbot (2016) [9] developed a scale of attachment to phones that taps into attachment dynamics. Their results showed a parallel between self-reported attachment style and the emotional bond connecting to one’s smartphone. Individuals with higher self-reported anxious attachment tended to perceive their phones as a “a refuge”, with increased discomfort when separated from it. Although with a weaker association avoidant individuals reported a sense of relief when “freed” from the device, which they experienced as “a burden”. Expectedly, the perception of one’s smartphone as a “refuge” was also strongly associated with measures of phone addiction. The fact that avoidant attachment was associated with feeling overwhelmed by the use of the smartphone is consistent with studies showing a negative association between avoidant style and smartphone, text messaging and SNS use [45].

### Self-regulatory functions of smartphone use

If the smartphone can be used as a putative attachment object, it is likely that it will enable self-regulatory functions for the insecurely attached individual. Given that adult attachment styles are likely to affect smartphone use indirectly [12], it is important to disentangle the mechanisms through which attachment affects the way people use new technologies. Given the greater vulnerability that individuals with higher attachment insecurity have in the areas of emotion regulation and self-esteem [46, 47], it is probable that they rely on their phone to deal with their difficulties to a greater extent. This pattern is also suggested by previous investigations in adults showing that individuals with high attachment anxiety use Facebook more frequently, especially when experiencing negative emotions [48]. The possibility that smartphones and SNS usage serves self-regulatory purposes and help overcome emotional difficulties is also suggested by longitudinal studies showing that chronically stressed as well as depressed individuals, tend to rely on online video gaming and smartphone use as a coping strategy to deal with unpleasant emotional states [49, 50].

As a form of emotion dysregulation, alexythimia (i.e., difficulties in recognizing and verbalizing one own’s emotions) is particularly relevant to understanding the connection between unsecure attachment and problematic (or more intense) smartphone use. Research suggests that individuals higher in anxious and avoidant attachment are more alexithymic [51], and that insercurely attached individuals tend to present a more elevated use of Facebook as a way to manage negative feelings [52]. Alexithymia has documented associations with unpleasant affective states such as depression and anxiety [53, 54] and is considered a risk factor for smartphone addiction [55, 56]. Considering these findings together, it is possible to argue that the activities performed on smartphones might fulfill a “mood alteration” function and serve as external regulators of negative emotional states [57].

### Limitations of extant research: toward a more comprehensive perspective

Studies that adopted such an integrative framework including interpersonal characteristics (e.g., attachment styles) with potential psychological mediators (e.g., alexithymia and difficulties in self-esteem) have been limited in two ways. First, although the association between alexithymia, attachment anxiety, and smartphone addiction has been established by a recent meta-analysis [58], the focus on smartphone addiction rather than on the affective experience deriving from using the smartphone (i.e., attachment to phone) limits the generalizability of the findings to adaptive uses of the phone and the possibility to understand if interpersonal attachment styles generalize to everyday use objects such as smartphones. Second, the combined role of these factors in determining smartphone and SNS use behavior is not generally investigated. In particular, although previous studies highlight the importance of SNS use for individuals with low self-esteem [59], it is likely that a combination of different factors, including problems in emotion regulation and alexithymia, contribute to digital behavior [60].

Beyond attachment, research on interpersonal functioning has also focused on interpersonal dependency, a construct related to, yet conceptually and empirically distinct from attachment [61, 62]. Healthy Dependency (HD) refers to the ability to appropriately seek for help and guidance when needed, without feeling guilty or ashamed [13]. Conversely, excessive/pervasive reliance on others (i.e., Destructive Overdependence: DO), and the inability to lean on others for support even when needed (i.e., Dysfunctional Detachment: DD) lead to more negative outcomes. High levels of DO and DD have been consistently linked to increased risks for psychological and physical difficulties across different populations and cultures [17, 63, 64].

The negative outcomes of maladaptive dependency (i.e., DO and DD) include poor self-esteem, alexithymia, and depression [17, 63] which, as discussed above, may in turn lead to maladaptive smartphone and SNS use. Attachment patterns are expressed in consistent ways across different relationships and reinforced by a particular individual (i.e., attachment figure). In contrast, dependency-related behaviors entail a broad array of self-presentation styles that are tailored to situational constraints and demands [65]. Similarly, behavioral manifestations of dependency are triggered by cues from an array of people with whom the individual might interact, regardless of any potential resemblance of that person to internal representations of early caregivers [63, 66, 67]. In sum, interpersonal dependency is a promising and still uninvestigated factor that could illuminate the pathways to maladaptive smartphone and SNS use.

Finally, a series of demographic factors represent a risk for smartphone and SNS maladaptive use and are thus worthy of consideration for a comprehensive understanding of the pathways leading to maladaptive digital behaviors. These include younger age [68], single marital status [69, 70], and gender [71, 72]. A literature review [73] showed that problematic Internet use is especially frequent in both adolescent and emerging adults (i.e., 19 years and older), whereas smartphone addiction is more relevant for younger adolescents compared with emerging adults [19]. Regarding age, it should be noted that although smartphone use is typically widespread among younger individuals, the “smartphone revolution” involved also older populations. Smartphone ownership has risen from the 10% for older adults in 2011 to 61% in 2021 [74], and the device has potential to help the elderly in several everyday activities, most importantly allowing them to overcome their physical limitations to connect digitally with family and friends [75, 76].

The contribution of single marital status to maladaptive smartphone use may be multifactorial, but could be partly explained by the fact that SNS is used to cope with potential feelings of loneliness and to engage in partner search activities [69]. The effect of gender on smartphone and SNS use behavior is more controversial. Some studies show that women tend to spend more time on SNS and female gender has been identified as a risk factor for problematic phone use [71, 77]. However, evidence suggests that males are more prone to develop Internet addiction and that relationship status has an impact on SNS activity of males, but little effect on the activity of females [71, 78].

### The present study

Grounded in the emerging literature on attachment and smartphone use, this paper presents a study testing an integrative model of attachment to smartphone through the lens of individual interpersonal functioning. The study has three specific aims: (1) clarifying the pathways connecting adult attachment styles to the emotional bond that individuals develop with their smartphone, and to the intensity of SNS use, (2) investigating the contribution of interpersonal dependency patterns in the emotional bond individuals develop with their smartphone, and in the intensity of SNS use, and (3) illuminating the psychological processes through which attachment style can affect smartphone related outcomes. As noted earlier, although youths are prime consumers of smartphone technology, it is increasingly used by individuals well into middle and later adulthood as well [79]. Therefore, this study includes individuals across different age groups.

Our main hypothesis is that people enact their interpersonal dependency patterns and attachment styles in their phone use, with anxious attachment and overdependence contributing to higher affective investment towards the device (i.e., perceiving it as “a refuge”), and higher intensity of SNS. In contrast, avoidant attachment and dysfunctional detachment will lead to a more “distant” relationship with the phone (i.e., perceiving it as “a burden”), as well as to lower SNS intensity of use. Even though empirical evidence on the structure of the attachment-interpersonal dependency relationship is limited, childhood attachment has been conceptualized as an antecedent of adolescent and adult dependency [80]. As Gewirtz (1973) [67], and Bornstein (2005) [81] have noted, attachment styles are grounded in early interactions with caregivers, whereas dependency represents a more generalized personality style that is expressed in a broad array of interpersonal relationships. Accordingly, this study modeled attachment as the main predictor of maladaptive phone use, whereas interpersonal dependency was conceptualized as a mediator of this relationship. The contributions of self-esteem and alexithymia were also accounted for in terms of potential mediation effects, in light of their known associations with attachment and interpersonal dependency [17] which, in turn, are related to social media and smartphone use [59, 82].

**Fig. 1:**
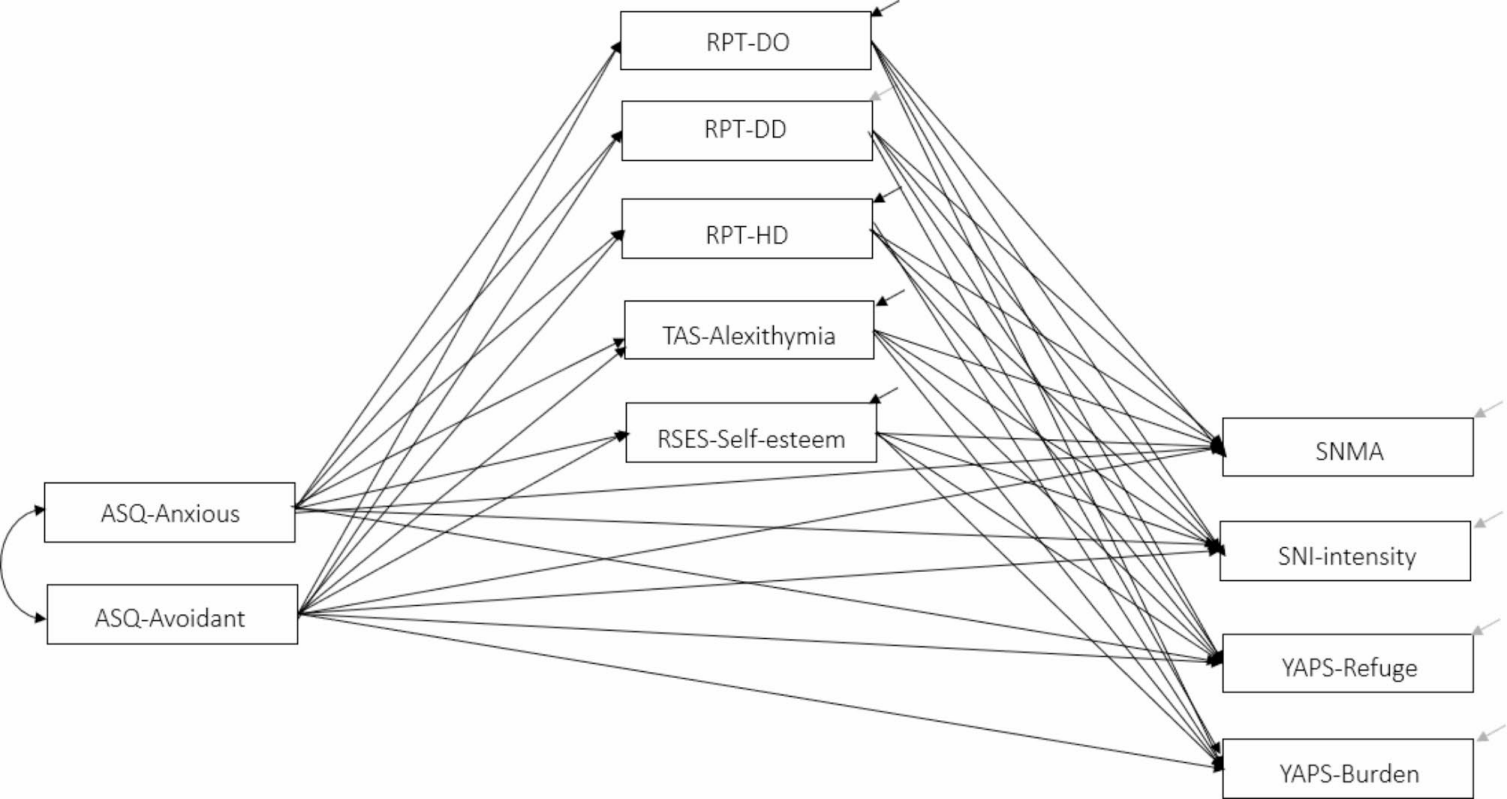
Hypothesized path model *Legend*. RPT-DO = Relationship Profile Test – Destructive Overdependence, RPT-DD = Relationship Profile Test – Dysfunctional Detachment, RPT-HD = Relationship Profile Test – Healthy Dependency; ASQ-ANX = Attachment Style Questionnaire – Anxious; ASQ-AVD = Attachment Style Questionnaire – Avoidant; YAPS-Refuge = Young Adult Attachment to Phone – Refuge, YAPS-Burden = Young Adult Attachment to Phone – Burden; SNI = Social Network Intensity, SNMA = Social Network Mobile Applications; TAS-Alexithymia = Toronto Alexithymia Scale total score; RSES-Self-esteem = Rosenberg Self-esteem Scale total score

Given the significant role played by single marital status in the consumption behavior of smartphone and SNS [69] as well as interpersonal dependency patterns (per theoretical expectations), marital status was modeled as a moderator of the relationships between interpersonal characteristics and smartphone/SNS use. Specifically, we hypothesized that the strength of the association between interpersonal characteristics and smartphone/SNS consumption would vary as a function of marital status, whereby single participants would report higher use of their smartphones and SNS than participants in a relationship. Finally, the potential confounding effect of demographic variables including age [68] and gender was also accounted for (e.g., [55]). The resulting hypothesized model is depicted in Fig. 1. To the best of the authors’ knowledge, this is the first study that studies attachment to phone and intensity of SNS use starting from attachment styles and potential mediators such as interpersonal dependency, alexithymia, and level of self-esteem. As such, the present study has an exploratory set-up.

## Method

### Participants

A total of 376 nonclinical participants from the Italian population was involved in the study. Participants were recruited from university courses in psychology and other disciplines, and through snowball sampling technique to enrich the sociodemographic composition of the sample and increase the generalizability of the study findings. Seven participants have been excluded from the analyses because they returned incomplete consent forms or an empty research battery. To minimize response bias due to social desirability and inaccuracy in responding, 26 participants were excluded from the analyses as they obtained above threshold scores on the Virtue scale (invalid protocol if score equal to or greater than 3) and one based on the Infrequency scale (invalid protocol if score equal to or greater than 4) of the Elemental Psychopathy Assessment [83]. The final sample included 341 participants (57.2% female; age range = 18–77, *M* = 35.37, *SD* = 14.50 years). In terms of marital status: 35.8% were single, 30% married, 19.7% in a relationship, 7.9% living together, 1.8% separated, 1.5% divorced, and 3.3% undeclared. Based on a statistical power analysis performed to estimate required sample size, the final sample size was adequate to detect expected small to medium effect sizes (d = 0.1 to 0.3)^1^ in correlations between target variables such as attachment style and attachment to phone based on previous studies [9].

### Measures

A series of self-report questionnaires was used to measure the variables of interest and are presented here following their order specified in the proposed model (i.e., predictors, mediators, and outcome variables).

**Attachment Style Questionnaire** (ASQ; [84, 85]. The ASQ is one of the most used self-report instruments to assess characteristics of attachment in adults. The Italian version is psychometrically comparable to the original version, and comprises 40 items rated on a 6-point scale ranging from *Totally Disagree* to *Totally Agree*). The questionnaire measures five subdimensions of adult attachment: Confidence, Discomfort with Closeness, Need for Approval, Preoccupation with Relationships, and Relationships as Secondary. Good reliability and validity evidence for the ASQ scores have been provided for both English and Italian versions of the instrument. For the present study, we computed the two summary indices reflecting the Anxious (average of Preoccupation with Relationships and Need for Approval), and Avoidant types of unsecure attachment (average of Discomfort with Closeness and Relationships as Secondary [86], and internal consistencies were satisfactory in the sample (Cronbach alpha = 0.77 and 0.82 respectively).

**Relationship Profile Test** (RPT; 17,45). The RPT is a self-report questionnaire with 30 items describing different manifestations of interpersonal dependency and rated on a 5-point Likert scale spanning from 1 (*Not at all true of me*) and 5 (*Very much true of me*). Consistent with the original version of the RPT, the RPT-I yields separate scores for Destructive Overdependence (DO), Dysfunctional Detachment (DD), and Healthy Dependency (HD). The RPT-I maintains the same format of the original instrument in terms of item content, order, and rating scales, and showed adequate criterion validity and temporal stability. In the present sample, internal consistency for DO was good (*α =* 0.74), acceptable for DD (*α* = 0.65), but limited for HD (*α =* 0.57). Although not optimal, this pattern is consistent with previous research documenting a lower internal consistency compared to DO and DD, and potentially suggesting that HD is a broader, more heterogeneous construct [17, 87].

**Rosenberg Self-Esteem Scale** (RSES; [88]. The RSES is a 10-item self-report measure that uses a 7-point response format ranging from 1 (*Strongly disagree*) to 4 (*Strongly agree*) which assesses global trait self-esteem. The Italian version used in this study [88] is widely used in research settings [89]. In the present study, internal consistency for the RSES scores was good (*α* = 0.85).

**Toronto Alexithymia Scale** (TAS-20; [90]. The TAS-20 is a self-report questionnaire to assess alexithymia and was used in this research as a proxy for emotional deficits in identifying and expressing one’s own emotions. The 20 items of the instrument are rated using a 5-point Likert scale ranging from 1 (*I totally disagree*) to 5 (*I totally agree*) and measure three different aspects of alexithymia: Difficulties Identifying Feelings, Difficulties Describing Feelings, and Externally-Oriented Thinking. The Italian version of the TAS-20 has yielded good internal consistency and test-retest reliability in both clinical and non-clinical populations [91]. In this study, the total alexithymia scale score, calculated using the 20 TAS items, yielded good internal consistency (*α* = 0.77).

**Young Adult Attachment to Phone Scale** (YAPS; 9). The YAPS self-report questionnaire was developed to assess attachment to phone through six items to be rated on a five-point Likert scale going from 1 (*Does not describe me at all*), to 5 (*Describes me perfectly*). It conceptualizes the individual’s relationship with their phone along the two distinct dimensions of Refuge (i.e., perceiving the object as a secure base, feeling safe when close to it and anxious when separated) and Burden (i.e., feeling overwhelmed by the presence of the phone and attempting to separate from it). The YAPS yields strong psychometric properties in terms of reliability, factorial validity, and criterion validity with relevant constructs [9]. In order to obtain an equivalent Italian version of the YAPS, a back-translation procedure was conducted following recommended guidelines [92, 93]. In the present sample, internal consistency for the Refuge and Burden scales fell in the good (*α =* 0.76) and acceptable range (*α =* 0.65) respectively.

**Social Network Intensity and Social Network Access via Mobile Phone Applications** (SNI, SNMA; 20). The SNI and SNMA are a set of items (six and five respectively) specifically developed to assess the relevance of SNS in the respondent’s life, in terms of frequency of use, personal involvement, and the use of SNS via mobile phone. As in Trub and Barbot’s[9] study, one item was added to inquire if social networking is one of the main activities participants did with their phone, and items were answered with a 5- point scale, ranging from “strongly disagree” to “strongly agree”. The SNI and SNMA demonstrated good internal consistency and discriminant validity, as well as utility in collecting information relevant in the context of mobile phone addiction. A back-translation procedure was carried out following recommended guidelines [92, 93] to translate the questionnaire in Italian. In the present sample, internal consistency was excellent for both scales (*α* = 0.90 for SNI and 0.97 for SNMA).

### Procedure

Each participant was provided with the consent form presenting the study and the age requirement for participation (i.e., 18 years or older) and those who provided a valid written informed consent received the research survey. The battery included sociodemographic questions and the six self-report questionnaires. Each participant filled in the survey on paper individually. The study was approved by the Institutional Review Board of University of Milano Bicocca (Protocol No. 157). Participants did not receive any form of compensation to take part to this research.

### Data analyses

Data checks were first performed to examine and treat missing values on all study variables, and screen for multivariate outliers and non-normality of all variables used in planned analyses. The amount of missing data was minimal, averaging 2.1% (range = 0.8 − 2.4%), with a pattern of missingness completely random (Little’s MCAR test: χ^2^_[*df* = 34]_ = 23.46, *p* = .913). To maximize statistical power in planned analyses, missing values were inputted using Bayesian multiple imputations (each using 10,000 Markov chain Monte Carlo observations). The complete dataset was screened for multivariate normality, suggesting violation of this assumption (Mardia multivariate kurtosis value 10.34, *z* = 3.01, *p* < .003). After deletion of 11 influential multivariate outliers (*p* < .05), the assumption of multivariate normality required for planned analyses was met (Mardia = 3.05, *z* = 1.40, *p* = .16).

Next, a multivariate analysis of variance with covariate (MANCOVA) was conducted to compare mean differences on all predictor and outcome variables specified in our model as a function of the relationship status (single vs. not single), and while accounting for the potential confounding effect of age and gender (i.e., modeled as covariates). Finally, the hypothesized path model (Fig. 1) of the relationship between attachment (ASQ^2^) and media use (SNI, SNMA, YAPS), as mediated by the distinct dependency and detachment styles (RPT), self-esteem (RSES), and alexithymia (TAS), and moderated by relationship status (i.e., hypothesis that the strength of these associations varies according to the relationship status) was then tested using multi-group path analysis implemented in the Structural Equation Modeling framework. The model also incorporated age and gender as covariates to eliminate the potential influence of these background variables. This analytic strategy was meant to detect model parameters of interest (here, beta weights of the direct and indirect effects) that significantly differ as a function of the values of the moderator (i.e., single vs. not single). To assess model fit, established benchmarks were used [94], including the non-significance of the χ^2^ test of fit, the Tucker-Lewis (TLI) and comparative fit index (CFI) > 0.95, the standardized root mean square residual (SRMR) ≤ 0.08, and root mean square error of approximation (RMSEA) ≤ 0.05. All estimates were computed using Maximum Likelihood, and 95% bias-correct confidence intervals using 500 bootstrap samples. The analyses were conducted using SPSS 27 and all path analyses were tested using SPSS AMOS version 22 [95, 96].

## Results

### Mean differences

Zero order Pearson’s correlations between attachment styles, interpersonal dependency dimensions, attachment to phone and SNS use are presented in Supplement Table 1. The omnibus test of the MANCOVA detected a significant effect of the tested model at risk *α* 0.05. Specifically, model estimates suggested an overall effect of age (F_[11, 305]_ = 10.63, *p* < .001, *η*² = 0.28) and gender (F_[11, 305]_ = 3.92, *p* < .001, *η*² = 0.12) on the variables under study, according to the relationship status (F_[11, 305 ]_ = 1.85, *p* < .05, *η*² = 0.06). Specifically, univariate effects of gender were all negligible (*η*² < 0.04), so were most effects of age. However, age had a moderate effect on SNI (*p* < .001, *η*² = 0.16) and SNMA (*p* < .001, *η*² = 0.22) Controlling for the influence of gender and age, there was still a significant effect of the relationship status on all predictor variables of interest (dependency, attachment, self-esteem and alexithymia), but not the outcome variables (i.e., social network intensity and social network use on mobile applications). Table 1 shows the estimated marginal means (i.e., controlling for age and gender) and univariate *F* test statistics of estimated marginal means, as a function of relationship status. As shown, the larger effect was observed for RPT-Healthy Dependency (HD), whereby single participants scored significantly lower than those who were non-single. Overall, the differences observed across variables were marginal (partial *h*² < 0.04).

**Table 1:** Estimated means and univariate comparisons for all variables of interest as a function of the relationship status

Scale	Estimated means (Std. Err)		Univariate Comparisons ^a^		
	Not single	Single	*F (1, 318)*	*p*	η² ^b^
***ASQ-Anxious attachment***	**50.01 (0.75)**	**53.41 (1.01)**	**6.45**	**0.012**	**0.02**
***ASQ-Avoidant attachment***	**44.31 (0.79)**	**47.80 (1.07)**	**6.11**	**0.014**	**0.02**
***RPT-Destructive Overdependence (DO)***	**2.52 (0.04)**	**2.70 (0.06)**	**5.14**	**0.024**	**0.02**
***RPT-Dysfunctional Detachment (DD)***	**3.17 (0.04)**	**3.37 (0.06)**	**7.62**	**0.006**	**0.02**
***RPT-Healthy Dependency (HD)***	**3.31 (0.04)**	**3.09 (0.05)**	**11.83**	**< 0.001**	**0.04**
***RSES-Self-Esteem***	**3.40 (0.04)**	**3.21 (0.05)**	**8.01**	**0.005**	**0.02**
***TAS-Alexithymia***	**2.34 (0.04)**	**2.51 (0.06)**	**5.32**	**0.022**	**0.02**
*YAPS-Attachment to phone - Refuge*	2.44 (0.07)	2.48 (0.09)	0.16	0.687	0.00
*YAPS-Attachment to phone - Burden*	2.55 (0.07)	2.60 (0.09)	0.18	0.672	0.00
*SNI-Social Network Intensity*	2.67 (0.08)	2.82 (0.11)	1.20	0.274	0.00
*SNMA-Social Network on Mobile Applications*	3.42 (0.09)	3.54 (0.12)	0.61	0.435	0.00

*Note*. ^a^ = Univariate ANOVAs comparing the Single vs. not Single groups controlling for age (34.91) and gender (1.43). ^b^ = Partial Eta squared. All significant results (i.,e., p < .05) are written in bolded format

### Path analyses

**Fig. 2:**
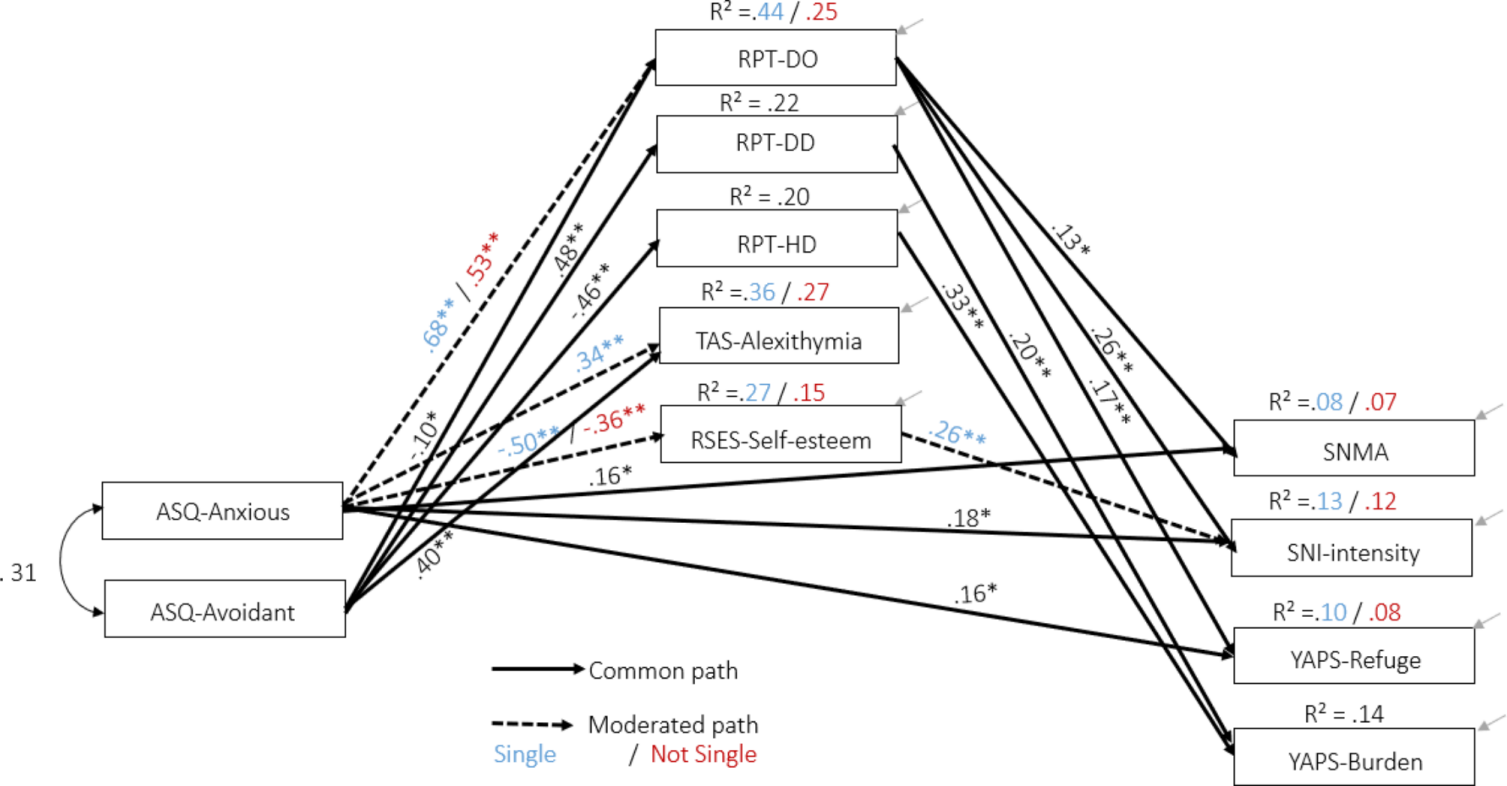
Summarized path model with all standardized beta obtained for the partially equivalent model *Legend*. RPT-DO = Relationship Profile Test – Destructive Overdependence, RPT-DD = Relationship Profile Test – Dysfunctional Detachment, RPT-HD = Relationship Profile Test – Healthy Dependency; ASQ-ANX = Attachment Style Questionnaire – Anxious; ASQ-AVD = Attachment Style Questionnaire – Avoidant; YAPS-Refuge = Young Adult Attachment to Phone – Refuge, YAPS-Burden = Young Adult Attachment to Phone – Burden; SNI = Social Network Intensity, SNMA = Social Network Mobile Applications; TAS-Alexithymia = Toronto Alexithymia Scale total score; RSES-Self-esteem = Rosenberg Self-esteem Scale total score

A *baseline* model was first specified such that all model parameters other than the beta weights were set to be equal across both *single* (n = 129) and *non-single* (n = 190) groups (i.e., equal means, intercepts, error variances and covariances). This rather constraining model was used to test the assumption that all model parameters (other than the beta weights under focus here) could be considered equivalent across groups, such that common parameters estimates across groups could be obtained. This model returned an adequate fit to the data (χ^2^_[df]_ = 84.7_[53]_, *p* < .01; χ^2/^_[df]_ = 1.60; CFI = 0.974; RMSEA_[95%CI]_ = 0.043 [0.025 − 0.060]). It was compared to a *fully equivalent* model according to which all parameters were set to be equal (i.e., adding the constraint of beta weights equivalence across both groups). This model was associated with an overall satisfactory fit to the data (χ^2^_[df]_ = 139.3_[91]_, *p* < .001; χ^2/^_[df]_ = 1.53; CFI = 0.961; RMSEA_[95%CI]_ = 0.041 [0.027 − 0.054]) but which statistically worsen significantly compared to the baseline model (Δχ^2^_[38]_ = 54.62, *p* = .04. ΔCFI = − 0.015). Therefore, based on the *fully equivalent* model, we derived a *partially equivalent* model from which equivalence constraints were relaxed for paths that differed significantly across groups (i.e., moderated paths). Using critical ratio (CR) for differences in model parameters, beta weight estimates that were not significantly different between groups (CR < 1.96, *p* = .05) were set to be equal, whereas those that differed significantly (CR > 1.96, *p* = .05) were freely estimated for each group. In all, four beta weights estimates were not equivalent across groups, namely: ASQ-Anxious attachment → RPT-Destructive Overdependence (CR = -3.10), ASQ-Anxious attachment → RSES-Self-esteem (CR = 2.54), ASQ-Anxious attachment → TAS-Alexithymia (CR = -3.10), RSES-Self-esteem → SNI-Social Network Intensity (CR = -2.48). The resulting model fit of this *partially equivalent* model was satisfactory (χ^2^_[df]_ = 119.0_[87]_, *p* < .05; χ^2/^_[df]_ = 1.37; CFI = 0.974; RMSEA_[95%CI]_ = 0.034 [0.016 − 0.048]), improved significantly over the *fully equivalent* model (Δχ^2^_[4]_ = 20.31, *p* = .001; ΔCFI = 0.015) and did not significantly differ from the *baseline* model (Δχ^2^_[34]_ = 34.43, *p* = .44; ΔCFI = 0.000). This final model is showed in Fig. 2.

Table 2 presents the unstandardized and standardized estimates and their corresponding bias-corrected bootstrapped 95% confidence interval for all direct effects, and Table 3 presents information for all indirect effects. Figure 1 displays the summarized path model with all standardized beta obtained for the partially equivalent model. Together, the overall model explained a small to moderate portion of the total variance in all media use variables, ranging from R² = 0.07 (SNMA among non-single participants) to R² = 0.14 (YAPS- Burden, in both groups) and several effects in the hypothesized direction were found. The confounding effect of age and gender (modeled as covariate of all predictor, mediator, and outcome variables in the model) was limited, with age being only meaningfully (i.e., *r* > .25) related to SNI ( *r = −* .42).

**Table 2:** Beta Weights Estimates for the Direct Effects in the Tested Model

Direct Paths	Unstandardized				Standardized			
	Est.	S.E.	95%CI_[L,U]_	*p*	Est.	S.E.	95%CI_[L,U]_	*p*
**ASQ-Anxious attachment -> RPT-Destructive Overdependence (single)**	**0.424**	**0.041**	**0.342, 0.506**	**0.003**	**0.684**	**0.043**	**0.594, 0.760**	**0.003**
**ASQ-Anxious attachment -> RPT-Destructive Overdependence (not single)**	**0.284**	**0.038**	**0.206, 0.356**	**0.003**	**0.527**	**0.058**	**0.409, 0.629**	**0.003**
**ASQ-Avoidant attachment -> RPT-Destructive Overdependence (all)**	**− 0.065**	**0.029**	**− 0.124, − 0.007**	**0.026**	**− 0.098**	**0.045**	**− 0.186, − 0.011**	**0.027**
ASQ-Anxious attachment -> RPT-Healthy Dependency (HD) (all)	0.035	0.025	− 0.021, 0.080	0.215	0.076	0.055	− 0.042, 0.175	0.200
ASQ-Anxious attachment -> RPT-Dysfunctional Detachment (all)	− 0.020	0.027	− 0.074, 0.036	0.577	− 0.039	0.054	− 0.142, 0.065	0.557
**ASQ-Avoidant attachment -> RPT-Healthy Dependency (HD) (all)**	**− 0.227**	**0.026**	**− 0.275, − 0.180**	**0.007**	**− 0.461**	**0.047**	**− 0.548, − 0.364**	**0.007**
**ASQ-Avoidant attachment -> RPT-Dysfunctional Detachment (all)**	**0.265**	**0.029**	**0.197, 0.329**	**0.004**	**0.475**	**0.052**	**0.370, 0.576**	**0.004**
**ASQ-Anxious attachment -> RSESScore (single)**	**− 0.025**	**0.004**	**− 0.032, − 0.015**	**0.006**	**− 0.501**	**0.070**	**− 0.628, − 0.333**	**0.005**
**ASQ-Anxious attachment -> RSES_Score (not single)**	**− 0.017**	**0.003**	**− 0.022, − 0.011**	**0.004**	**− 0.364**	**0.063**	**− 0.427, − 0.246**	**0.004**
**ASQ-Anxious attachment -> TAS-Alexithymia (single)**	**0.018**	**0.004**	**0.008, 0.029**	**0.004**	**0.343**	**0.087**	**0.156, 0.500**	**0.005**
**ASQ-Anxious attachment -> TAS-Alexithymia (not single)**	**0.007**	**0.003**	**0.000, 0.012**	**0.048**	**0.132**	**0.058**	**− 0.007, 0.237**	**0.053**
ASQ-Avoidant attachment -> RSES_Score (all)	− 0.003	0.003	− 0.008, 0.003	0.330	− 0.056	0.052	− 0.150, 0.054	0.340
**ASQ-Avoidant attachment -> TAS-Alexithymia (all)**	**0.022**	**0.003**	**0.018, 0.028**	**0.003**	**0.395**	**0.049**	**0.307, 0.493**	**0.003**
**RPT-Destructive Overdependence -> YAPS- Refuge (all)**	**0.024**	**0.010**	**0.005, 0.045**	**0.008**	**0.171**	**0.073**	**0.034, 0.314**	**0.008**
**RPT-Destructive Overdependence -> SNI- Intensity (all)**	**0.044**	**0.012**	**0.022, 0.066**	**0.004**	**0.260**	**0.065**	**0.118, 0.372**	**0.005**
**RPT-Destructive Overdependence -> SNMA (all)**	**0.028**	**0.015**	**0.001, 0.054**	**0.048**	**0.133**	**0.064**	**0.003, 0.253**	**0.048**
**ASQ-Anxious attachment -> SNMA (all)**	**0.021**	**0.009**	**0.005, 0.038**	**0.019**	**0.164**	**0.068**	**0.033, 0.293**	**0.018**
**ASQ-Anxious attachment -> SNI- Intensity (all)**	**0.019**	**0.007**	**0.003, 0.032**	**0.02**	**0.179**	**0.071**	**0.036, 0.320**	**0.016**
**ASQ-Anxious attachment -> YAPS- Refuge (all)**	**0.014**	**0.006**	**0.001, 0.026**	**0.037**	**0.160**	**0.072**	**0.020, 0.299**	**0.033**
ASQ-Avoidant attachment -> SNMA (all)	− 0.003	0.011	− 0.023, 0.020	0.848	− 0.020	0.079	− 0.167, 0.145	0.848
ASQ-Avoidant attachment -> SNI- Intensity (all)	0.003	0.008	− 0.013, 0.020	0.644	0.031	0.076	− 0.117, 0.179	0.637
ASQ-Avoidant attachment -> YAPS- Refuge (all)	0.002	0.007	− 0.012, 0.016	0.730	0.026	0.078	− 0.128, 0.174	0.730
ASQ-Anxious attachment -> YAPS- Burden (all)	− 0.006	0.006	− 0.018, 0.008	0.479	− 0.068	0.079	− 0.208, 0.093	0.467
ASQ-Avoidant attachment -> YAPS- Burden (all)	0.012	0.007	0.000, 0.025	0.058	0.131	0.070	− 0.003, 0.278	0.055
RPT-Destructive Overdependence -> YAPS- Burden (all)	− 0.001	0.010	− 0.023, 0.018	0.829	− 0.006	0.077	− 0.172, 0.132	0.837
RPT-Dysfunctional Detachment -> SNMA (all)	− 0.026	0.015	− 0.055, 0.009	0.108	− 0.104	0.067	− 0.217, 0.036	0.115
RPT-Dysfunctional Detachment -> SNI- Intensity (all)	− 0.010	0.012	− 0.032, 0.017	0.406	− 0.051	0.062	− 0.164, 0.083	0.418
RPT-Dysfunctional Detachment -> YAPS- Refuge (all)	0.001	0.010	− 0.019, 0.024	0.885	0.007	0.065	− 0.117, 0.144	0.893
**RPT-Dysfunctional Detachment -> YAPS- Burden (all)**	**0.032**	**0.010**	**0.013, 0.052**	**0.003**	**0.200**	**0.065**	**0.088, 0.332**	**0.002**
RPT-Healthy Dependency (HD) -> SNMA (all)	0.004	0.018	− 0.032, 0.039	0.791	0.013	0.065	− 0.113, 0.137	0.784
RPT-Healthy Dependency (HD) -> SNI- Intensity (all)	0.010	0.014	− 0.018, 0.040	0.472	0.043	0.066	− 0.084, 0.173	0.491
RPT-Healthy Dependency (HD) -> YAPS- Refuge (all)	− 0.004	0.012	− 0.029, 0.021	0.778	− 0.022	0.067	− 0.157, 0.117	0.801
**RPT-Healthy Dependency (HD) -> YAPS- Burden (all)**	**0.060**	**0.011**	**0.039, 0.081**	**0.006**	**0.333**	**0.061**	**0.225, 0.443**	**0.005**
RSES_Score -> SNMA (all)	− 0.012	0.185	− 0.384, 0.369	0.956	− 0.005	0.072	− 0.141, 0.144	0.955
**RSES_Score -> SNI- Intensity (single)**	**0.561**	**0.168**	**0.191, 0.889**	**0.006**	**0.261**	**0.084**	**0.081, 0.441**	**0.008**
RSES_Score -> SNI- Intensity (not single)	0.123	0.185	− 0.202, 0.489	0.496	0.053	0.08	− 0.09, 0.201	0.515
RSES_Score -> YAPS- Refuge (all)	0.025	0.122	− 0.213, 0.274	0.813	0.014	0.070	− 0.116, 0.151	0.805
RSES_Score -> YAPS- Burden (all)	− 0.183	0.115	− 0.441, 0.041	0.132	− 0.107	0.070	− 0.247, 0.036	0.151
TAS-Alexithymia -> SNMA (all)	− 0.009	0.170	− 0.366, 0.293	0.973	− 0.004	0.071	− 0.150, 0.121	0.989
TAS-Alexithymia -> SNI- Intensity (all)	0.092	0.134	− 0.151, 0.375	0.474	0.047	0.065	− 0.082, 0.175	0.474
TAS-Alexithymia -> YAPS- Refuge (all)	0.046	0.112	− 0.158, 0.262	0.626	0.029	0.067	− 0.101, 0.160	0.633
TAS-Alexithymia -> YAPS- Burden (all)	− 0.068	0.106	− 0.284, 0.149	0.525	− 0.043	0.069	− 0.184, 0.093	0.525

*Note*. Est. = Estimated parameter value; S.E. = Standard Error of estimate. All significant results (i.,e., p < .05) are written in bolded format

**Table 3:** Beta Weights Estimates for the Indirect Effects in the Tested Model

Indirect Paths	Unstandardized				Standardized			
	Est.	S.E.	95%CI_[L,U]_	*p*	Est.	S.E.	95%CI_[L,U]_	*p*
ASQ-Anxious attachment -> SNMA (single)	0.013	0.007	− 0.002, 0.028	0.077	0.097	0.056	− 0.009, 0.212	0.067
**ASQ-Anxious attachment -> SNMA (not single)**	**0.009**	**0.005**	**0.001, 0.021**	**0.041**	**0.068**	**0.039**	**0.003, 0.159**	**0.042**
ASQ-Anxious attachment -> SNI- Intensity (single)	0.007	0.007	− 0.007, 0.021	0.257	0.068	0.063	− 0.068, 0.194	0.253
**ASQ-Anxious attachment -> SNI- Intensity (not single)**	**0.012**	**0.005**	**0.003, 0.022**	**0.004**	**0.111**	**0.042**	**0.032, 0.211**	**0.004**
ASQ-Anxious attachment -> YAPS- Refuge (single)	0.004	0.005	− 0.007, 0.015	0.448	0.052	0.065	− 0.089, 0.179	0.454
ASQ-Anxious attachment -> YAPS- Refuge (not single)	0.004	0.004	− 0.004, 0.01	0.410	0.057	0.046	− 0.053, 0.126	0.416
ASQ-Avoidant attachment -> SNMA (single)	− 0.010	0.007	− 0.025, 0.003	0.169	− 0.07	0.053	− 0.184, 0.025	0.172
ASQ-Avoidant attachment -> SNMA (not single)	− 0.010	0.007	− 0.025, 0.021	0.169	− 0.07	0.068	− 0.184, 0.025	0.172
ASQ-Avoidant attachment -> SNI- Intensity (single)	− 0.007	0.006	− 0.019, 0.004	0.147	− 0.066	0.052	− 0.166, 0.035	0.147
ASQ-Avoidant attachment -> SNI- Intensity (not single)	− 0.006	0.006	− 0.017, 0.022	0.220	− 0.055	0.111	− 0.154, 0.047	0.195
ASQ-Avoidant attachment -> YAPS- Refuge (single)	− 0.006	0.005	− 0.016, 0.004	0.245	− 0.069	0.057	− 0.177, 0.043	0.258
ASQ-Avoidant attachment -> YAPS- Refuge (not single)	− 0.006	0.005	− 0.016, 0.01	0.245	− 0.069	0.045	− 0.177, 0.043	0.258
ASQ-Anxious attachment -> YAPS- Burden (single)	0.010	0.005	0.000, 0.021	0.054	0.118	0.058	− 0.006, 0.24	0.060
ASQ-Anxious attachment -> YAPS- Burden (not single)	0.007	0.004	0.000, 0.014	0.063	0.052	0.043	− 0.005, 0.162	0.061
ASQ-Avoidant attachment -> YAPS- Burden (single)	0.001	0.005	− 0.008, 0.009	0.972	0.007	0.051	− 0.088, 0.101	0.988
ASQ-Avoidant attachment -> YAPS- Burden (not single)	0.001	0.005	− 0.008, 0.014	0.972	0.007	0.076	− 0.089, 0.102	0.988

*Note*. Est. = Estimated parameter value; S.E. = Standard Error of estimate. All significant results (i.,e., p < .05) are written in bolded format

First, the attachment variables were expectedly related to all mediators of interest, with differential effects of the ASQ-Anxious attachment according to the relationship status. Overall, ASQ-Anxious attachment related more strongly to mediators Destructive Overdependence, Alexithymia, and Self-Esteem among singles (*β* = 0.68, *p* = .003, *β* = 0.34, *p* = .005, *β* == − 0.50, *p* = .003, respectively), than non-singles (*β* = = 0.53, *p* = .005, *β* = = 0.13, *p* = .05, *β* = = − 0.36, *p* = .003, respectively). Associations between ASQ-Avoidant attachment and all mediators were equivalent according to the relationship status, with effects of moderate magnitude. Together, both attachment variables explained between 20% and 44% of the variance in all mediators under study.

In terms of the prediction of the type of affective bond developed by participants with their smartphone starting from adult attachment style and interpersonal patterns (i.e., features of interpersonal dependency), YAPS- Attachment to phone - Refuge was directly explained by ASQ-Anxious attachment (*β* = 0.160, *p* = .033) with no indirect influence through the mediators investigated (*β* = − 0.07, *p* = .26), and by RPT-DO through a direct common (i.e., same for both groups) effect (*β* = 0.171, *p* = .008). YAPS- Attachment to phone Burden was mainly directly explained by RPT-Healthy Dependency and Dysfunctional Detachment (*β* = 0.33, *p* = .005, and *β* = 0.20, *p* = .002, respectively), and by a negligible indirect influence of ASQ-Anxious attachment (indirect effect = 0.12, *p* = .06 among singles).

With regard to the prediction of all media use variables, SNI was directly predicted by a small effect of ASQ-Anxious attachment in both groups (i.e., not moderated by the relationship status; *β* = 0.18, *p* = .016). There was also an indirect effect of ASQ-Anxious attachment on SNI of small magnitude among the non-single participants (*β* = 0.111, *p* = .004). For singles, this indirect relationship was not observed *β* = 0.068, *p* = .253). SNMA was predicted by a partial mediation effect of ASQ-Anxious attachment, and it was moderated by the relationship status (common direct effect *β* = 0.16, *p* = .02; single indirect effect *β* = 0.097, *p* = .067, non-single indirect effect *β* = 0.068, *p* = .042 and by a direct effect of RPT- Destructive Overdependence on the whole group (*β* = 0.13, *p* = .05). Together the indirect and direct effect of ASQ-Anxious attachment on SNI combined into a total effect of moderate magnitude for both groups (for single *β* = 0.248, *p* = .004; for not-single *β* = 0.291, *p* = .004). SNI was also explained by RPT-DO through a direct effect of moderate magnitude and common to both groups of single and not-single participants (*β* = 0.26, *p* = .005). Finally, SNI was predicted by self-esteem as well, whereby a higher level of self-esteem predicts more SNS use, with an effect only detected among the single group (*β* = 0.26, *p* = .01). This relationship was not observed among non-single participants (*β* = 0.05, *p* = .51).

Taken together, these findings show that individual differences in interpersonal dependency and adult attachment style exert a significant influence on the affective bond that users develop with their smartphone and SNS intensity of use. These effects are mainly independent from the role of demographic confounding variables such as age and gender, although the relationship status plays a role in the case of SNS. In particular anxious attachment predicts higher levels of engagement with SNS and this effect works directly for the total group of participants and through the influence of mediators when analyzing the associations on the two relationship status groups. Overdependence, on the other hand, predicted more intense SNS use, independently from the relationship status.

## Discussion

Smartphones and SNS play an increasingly central role in everyday life, facilitating the implementation of a variety of tasks and allowing people to develop and maintain interpersonal relationships. Given this social function of such digital devices, and in light of their addictive potential [4], it is necessary to clarify the interpersonal patterns leading to adaptive versus maladaptive smartphone consumption. This study proposed a novel approach to this issue, combining the literature the self-regulatory function of smartphones and their applications [48, 56] with a new focus on interpersonal dimensions. We hypothesized that adult attachment style and interpersonal patterns (i.e., features of interpersonal dependency) generalize to the emotional bond with the device, interacting with other relevant psychological correlates including self-esteem and alexithymia, to affect smartphone and SNS consumption.

Overall, our findings are in line with the hypothesis concerning the influence of interpersonal styles on the individual’s relationship with their smartphone and use of SNS. As expected, anxious and avoidant attachment were related consistently and coherently to all interpersonal dependency variables, as well as to emotional deficit and self-esteem with effects of moderate to large magnitude. These associations were partly moderated by relationship status (i.e., single vs. non-single) whereby anxious attachment was a stronger predictor of destructive overdependence, alexithymia, and decreased self-esteem among singles compared to non-singles. In turn, anxious attachment both directly and indirectly contributed to maladaptive phone use (i.e., higher scores of SNMA, SNI, and YAPS-Refuge), especially among participants in a relationship. As such, anxious attachment had a stronger effect on maladaptive phone use for people who are in a relationship. This could mean that individuals with higher levels of anxious attachment and who are in a romantic relationship tend to use their smartphone to remain connected with their significant other and cope with their insecurity. Further evidence of a carryover effect of one’s relationship style to smartphone use was found in terms of the associations obtained between interpersonal dependency profiles and smartphone/SNS use. In particular, as expected, participants with higher interpersonal dependency reported a stronger emotional bond with their telephone, likely seen as a source of reassurance and “guidance” in navigating everyday life. Similarly, the more intense use of SNS reported by more interpersonally dependent individuals can be interpreted as a further attempt to satisfy their need of connectedness and relatedness with others. On the other hand, individuals who had higher levels of Healthy Dependency or Dysfunctional Detachment, and therefore were likely less in search of external confirmation and support, consistently identified the smartphone as a burden, feeling relieved when the device is out of reach.

Beyond the influence of adult attachment and interpersonal styles, other potentially relevant factors in the consumption of SNS have been investigated in the present study. Notably, greater use of SNS was predicted by higher levels of self-esteem among the single group, confirming and extending findings of previous studies on the potential implications of SNS use for processes of identity consolidation and self-enhancement. For example, Michikyan and colleagues [97] examined emerging adults’ presentation of their real self, ideal self, and false self on Facebook, in relation to their identity state and psychosocial well-being. The authors found a positive correlation between more coherent identity states and higher probability of presenting their real self on Facebook, and significant positive associations of a less coherent sense of the self and low self-esteem with false self-presentation on Facebook. Hence, it is possible that SNS are used more intensely to promote a favorable image of oneself, and that this behavior is more pronounced for single individuals.

Importantly, the observed findings significantly varied between single and non-single participants. Compared to those who are in a relationship, singles tended to report heightened psychological vulnerability. The latter took the form of higher levels of alexithymia and lower self-esteem, as well as more maladaptive interpersonal functioning indicated by higher avoidant and anxious attachment, dysfunctional detachment, destructive overdependence, and lower healthy dependency. This is in line with the long-known assumption that, in general, being in a romantic relationship is a protective factor against poorer mental and physical health [98]. However, the reverse pattern may occur as well, whereby self-reported psychological distress and difficulties might impede the engagement in significant romantic relationships, or such relationships themselves can be source of distress for the individual.

### Theoretical implications: Smartphones as objects of attachment

Beginning with Winnicott [41], the vast majority of writing on transitional objects has focused on children, and emphasized objects that are used to self-soothe in times of distress, and enable children to feel connected to caregivers even in the caregiver’s absence. In recent years, researchers have explored transitional object use in adults [99, 100]. Because people often utilize smartphones as tools for connecting with absent friends and distant family members, and as a method for self-soothing when anxious or distressed, researchers have begun to conceptualize smartphones as transitional objects as well—as objects of attachment that serve many of the same purposes as traditional transitional objects like security blankets and stuffed animals [9, 101]. For frequent users, smartphones not only reify continued connection with significant others who are not physically present, they also enable people to enact relationship dynamics from a distance (e.g., via text messaging; see [102]. In summarizing current thinking in this area, Cohen and Torous [103] noted,

The smartphone may function as a type of transitional object for an adult. The smartphone allows an individual to feel comforted and connected to others when feelings of physical pain, sadness, or other negative emotions arise. Digital connection is an immediate solution to discomfort, allowing individuals to feel connected to those who care about them (81; p.2169).

The present findings are consistent with this emerging conceptualization, and suggest that theoretical frameworks which have proved useful in understanding the intra- and interpersonal dynamics of transitional object use (e.g., attachment theory, object relations theory) may be useful in this context as well. Although it will be important to examine the ways in which the dynamics of adults’ use of transitional objects may vary across object categories (e.g., smartphones versus stuffed animals), it will also be important to continue to develop a unifying framework to contextualize and study transitional object use beyond childhood.

### Practical implications for minimizing maladaptive smartphone use

The present findings indicate that interpersonal styles characterized by anxious attachment and by overdependence on others, are potential risk factors for maladaptive smartphone and SNS use. It is possible that the smartphone is used, especially in moments of solitude, to manage unpleasant feelings such as loneliness and boredom [7, 48]. The fact that anxious attachment was also directly associated with higher intensity of social network use among both singles and individuals in a relationship suggests that SNS might be used as a mean to obtain security and minimize feelings of isolation and loneliness not necessarily based on the actual availability of a romantic partner.

Acknowledging the relevance of preoccupied adult attachment style in maladaptive media use has implications for assessment and treatment planning in the context of digital addiction. Our findings call for increased attention in the clinical assessment phase of the patient’s habits with potentially addictive devices, including smartphones, if indicators of anxious attachment are present. Findings from a meta-analysis [104] reveal how interpersonal patterns are not generally a point of attention in psychological interventions for smartphone addiction. Instead, such interventions often favor educational trainings aimed at improving academic motivation and efficient use of time, or consist of therapeutic actions targeted to the individual’s cognitions, conceptualized as the main source of maladaptive digital device consumption.

Further, our findings suggest that anxious attachment and overdependence may also interfere with effective use of smartphone-based technology to facilitate treatment compliance in medical settings, by causing people to overuse the smartphone when distressed, increasing health provider burden. Although telehealth has proven to be useful in alleviating health professionals’ stress from excessive workload in difficult times (e.g., during the Covid-19 pandemic; [105], it is possible that patients with anxious attachment, who also frequently present with somatoform disorders and require increased medical attention, might be more likely to exploit the healthcare provider’s resources by the search of constant communication [106]. It would be preferable for telemedicine and telepsychotherapy to be attuned to the insecurely attached and overdependent patient’s deep need of relatedness and their ways of obtaining it through frequent communication and implement a mindful enforcement of healthy professional boundaries.

### Limitations of the present study and future directions

The main limitation of the present study, which also suggests an avenue for future research, is the reliance on self-report measures of smartphone and SNS use. In future investigations, actual smartphone use could be assessed to exclude potential desirability or recall biases in participants’ reports of their digital behavior. Moreover, as it proved useful in studying attachment to smartphone but also other complex psychological constructs such as, for instance, narcissistic reactions to a self-esteem insult [107], research designs encompassing experimental manipulation of interpersonal dependency could help understand the effect of the user’s relationship style on the bond developed with the device. Secondly, the level of internal consistency detected for a few variables in this study was limited. As noted above, the complexity of the dimension of healthy dependency might be the reason of a lower internal consistency of the scale already documented in the literature. Furthermore, especially for shorter scales measuring complex construct, it is reasonable to consider Cronbach’s alpha of 0.60, to which can be approximated the lowest internal consistency coefficient registered for the scales used in this study, still acceptable [108] At any rate, does not challenge the validity of our findings, but it might have reduced the strength of the observed relationships.

Although the analysis of gender and age effects on smartphone and SNS use was not the object of the present study, we have systematically assessed their effect on the target outcome variables. Overall, we did not detect a relevant influence of these demographic variables on the predicted variables, coherently with the literature showing mixed effects for gender [55, 58, 59] and with the consideration that digital behaviors are a typical youth phenomenon which is however increasingly extending to the elderly as well. However, a promising line of future investigation would be a closer analysis of age and gender effects on smartphone attachment and SNS use.

There are also several strengths to this work. These include a large and demographically diverse community sample that extends to adulthood the study of attachment to phone previously investigated on smaller samples of college students [31]. This is particularly important because, although attachment styles are assumed to be mostly stable over time, they might undergo significant change during the life span, and in particular during periods of major developmental adaptation such as the transition to adulthood [109]. A second strength of the present study is the use of an integrative theoretical perspective that can help identify the mechanisms underlying human consumption of smartphone-based technology and mechanisms involved in their maladaptive use, emphasizing the role of insecure attachment style in affecting digital addiction through effects on other risk factors such as alexithymia.

Although a digital gap between more developed and emerging economies still exists, even in less technologically advanced countries, smartphone ownership rates have been steadily increasing in the last few years [44]. It is therefore highly relevant to pursue research on the factors explaining individual use of smartphones and SNS use across countries and cultures, to limit the appearance of maladaptive use behaviors. Attachment style is generally envisioned as a biologically determined behavioral system transversal to different cultures, whereas evidence of potential cultural influences on interpersonal dependency is still limited [17]. Given the potential cultural differences related to interpersonal dependency, it is important to assess cross-cultural generalizability of the present results to other geographical areas and cultural groups.

## Conclusions

In conclusion, the present study represents the first attempt to explore the contribution of the interpersonal dimension in smartphone and SNS use in combination with other known psychological factors such as self-esteem and emotional functioning. Findings support the idea of a parallel existing between attachment style and interpersonal dependency and the emotional bond connecting to one’s smartphone. Our results confirm that perceiving the device as a refuge from everyday difficulties is associated with more intense, and potentially maladaptive, SNS use. The importance of these interpersonal contributors to smartphone and SNS use might have the potential to contribute to the development of more individualized interventions for digital addictions.

### Electronic supplementary material

Below is the link to the electronic supplementary material.

Supplementary Material 1

## Data Availability

Data for this study have been uploaded by the authors on a secure public repository (i.e., www.osf.io) currently accessible through a private weblink. Such material will be made available upon reasonable request to the corresponding author.

## Abbreviations

SNS: Social Network Sites
PMPU: Problematic Mobile Phone Use
U.S.: United States of America
M: Mean
SD: Standard Deviation
TLI: Tucker-Lewis Index
CFI: Comparative Fit Index
SRMR: Standardized Root Mean Square Residual
RMSEA: Root Mean Square Error of Approximation
MANCOVA: Multivariate Analysis of Covariance
RPT-DO: Relationship Profile Test – Destructive Overdependence
RPT-DD: Relationship Profile Test – Dysfunctional Detachment
RPT-HD: Relationship Profile Test – Healthy Dependency
ASQ-ANX: Attachment Style Questionnaire – Anxious
ASQ-AVD: Attachment Style Questionnaire – Avoidant
YAPS-Refuge: Young Adult Attachment to Phone – Refuge
YAPS-Burden: Young Adult Attachment to Phone – Burden
SNI: Social Network Intensity
SNMA: Social Network Mobile Applications
TAS-Alexithymia: Toronto Alexithymia Scale total score
RSES-Self-esteem: Rosenberg Self-esteem Scale total score

## Footnotes

1 Power analysis was conducted with α = 0.05, with power = 0.80 and it showed a needed sample size of approximately 64 participants for point biserial correlation analyses, calculated in G*Power 3.1.9.7 [110]. The sample size of the present study was adequately powered also with a more stringent power set up (α = 0.05, 1-β = 0.95 needed sample size of approximately 111 participants).

2 To allow for a simpler and more interpretable model, and given that secure attachment correlated highly and negatively with the other two predictors (i.e., Avoidant and Anxious attachment, *rs* = − 0.705 and − 0.548 respectively, both ps < 0.01) in this sample, only the latter attachment styles were included as predictors.
