# Supplementary material for: The smartphone as a “significant other”: interpersonal dependency and attachment in maladaptive smartphone and social networks use
Source: BMC Psychol. 2023 Sep 28;11:296. doi: 10.1186/s40359-023-01339-4 (PMC10540499; doi:10.1186/s40359-023-01339-4)
Supplement: Supplementary file 1 — Supplementary Material 1 [file 40359_2023_1339_MOESM1_ESM.docx]

Supplementary table 1. Zero-order correlations between target variables.

|  |  | *Interpersonal dependency* | | | *Attachment style* | |
| --- | --- | --- | --- | --- | --- | --- |
|  |  |  | | |  | |
|  |  | RPT-DO | RPT-DD | RPT-HD | ASQ-ANX | ASQ-AVD |
|  |  |  |  |  |  |  |
| *Attachment to phone* | |  |  |  |  |  |
|  |  |  |  |  |  |  |
|  | YAPS-Refuge | .262** | .067 | -.072 | .291** | .137* |
|  | YAPS-Burden | .021 | .217** | .238** | -.021 | .018 |
|  | |  |  |  |  |  |
| *Social Network Use* | |  |  |  |  |  |
|  |  |  |  |  |  |  |
|  | SNI | .300** | .004 | .017 | .262** | .043 |
|  | SNMA | .226** | -.079 | -.007 | .239** | .004 |
|  |  |  |  |  |  |  |

*Note.* *p < .05; **p < .01; ***p < .001. RPT-DO = Relationship Profile Test – Destructive Overdependence, RPT-DD = Relationship Profile Test – Dysfunctional Detachment, RPT-HD = Relationship Profile Test – Healthy Dependency; ASQ-ANX = Attachment Style Questionnaire – Anxious; ASQ-AVD = Attachment Style Questionnaire – Avoidant; YAPS-Refuge = Young Adult Attachment to Phone – Refuge, YAPS-Burden = Young Adult Attachment to Phone – Burden; SNI = Social Network Intensity, SNMA = Social Network Mobile Applications.
